# Be Aware: Science Is Not Ready to Calculate the Antimicrobial Resistance Death due to Air Pollution

**DOI:** 10.3389/ijph.2023.1606497

**Published:** 2023-08-21

**Authors:** Nino Künzli, Meltem Kutlar Joss, Ron Kappeler

**Affiliations:** ^1^ Swiss Tropical and Public Health Institute, Allschwil, Switzerland; ^2^ University of Basel, Basel, Switzerland

**Keywords:** air pollution, WHO air quality guidelines, air quality, traffic-related air pollution, environmental health, microbial resistance, ecologic correlations

The new WHO air quality guideline values of fine particulate matter up to 2.5 μm in diameter call for a reduction of the annual mean standards from 10 to 5 μg per cubic meter ambient air [1]. The science of the association with death and disease is overwhelming and the estimated burden of more than 4 million deaths due to ambient PM2.5 stands on solid research. Methods for the estimation of the burden due to air pollution were developed in the mid 1990s and became inherent part of the global burden of disease in this century [2–4]. Meanwhile, assessments of burden of air pollution related death have become a global research industry, challenging authorities with seemingly contradicting results [5]. A recent paper [6] went a step further. As discussed in this editorial: far beyond the science one would need to derive a burden.

With reference to an estimated 1.27 million death attributable to antimicrobial resistance (AMR), authors claim that 0.48 Million—or 38% of all AMR deaths—to be due to ambient PM2.5 exposure. However, to estimate the burden attributable to some risk factor, it is of adamant importance to fulfill at least two criteria prior to embark on such quantitative procedures.

First, evidence should be established at least for a “likely causal” association between the risk factor and the outcome. In this case, genetic material carried on PM2.5 particles would cause the development of antibiotic resistance as the ultimate cause of premature death. Zhou et al. can only refer to a thin literature identifying AMR genes on ambient PM2.5 and hypotheses on the potential role of PM2.5 in its dissemination. Whether and how long-term inhalation of such genetic material, carried on ambient particles, can lead to the development of a clinically relevant—lethal—resistance against major antibiotics is not known at all. It is instead a hypothesis that waits to be tested with appropriate methods. The major unknowns are also emphasized by Jin et al. [7]—erroneously used by Zhou et al as rationale for their exercise. Jin’s abstract clearly states: “The ultimate question is the fate and consequences of inhalable antimicrobial resistance in interaction with microbiomes in healthy and diseased human airways, which would shed light on the role of AMR in viral-bacterial co-infections leading to acute and chronic respiratory diseases.”

Second, the quantification of the number of deaths (or of life years lost) attributable to a “likely causal” risk factor, requires knowledge about the so called “concentration response function” (CRF), i.e., the risk ratio per unit long-term exposure—in this case the association of PM2.5 particles carrying AMR genes- and mortality due to AMR. This CRF must originate from solid cohort data with individual estimates of the long-term exposure to such genetic material on PM2.5 and the occurrence of death due to antibiotic resistance. So far, such cohort studies have not been published at all. In the absence of this instrumental information—the CRF—it is obsolete to embark on the estimation of the causal burden.

Surprisingly, however, Zhou et al. fill this critical gap with a set of nationally aggregate data from 116 countries such as indicators for AMR and other macro data (e.g., GDP and health expenditures). One unit change in the national mean PM2.5 was statistically correlated with a 0.48% increase in the antibiotic resistance indicator developed as an indicator for the unknown death rates due to antibiotic resistance. This magic number was used as a proxy for the unavailable CRF to lead to the ultimate claim that compliance with the WHO AQG annual mean of 5 μg/m^3^ would reduce the death due to antibiotic resistance by 23.4%. The authors add the blunt causal statement that “These findings substantiate that PM2.5 is a primary factor driving global antibiotic resistance, suggesting global health could benefit from mitigating antibiotic resistance with PM2.5 control.”

We strongly disagree. Derivations of the burden in the absence of its most important input data are grossly misleading. Long ago, Brenner [8] has shown in a seminal paper how the use of purely ecologic correlations between aggregated long-term data to be fundamentally misleading. He used the well-established association between smoking and lung cancer death to demonstrate that cross-regional correlations of aggregated variables strongly depend on the—by default unknown—sensitivity and specificity of the aggregated input data. In the absence of individual level data it is impossible to even predict the direction and potential size of the uncontrollable biases. Despite far more “precise” smoking related aggregate data such as the regional prevalence of smoking—as compared to the complex derivation of an indicator of the national antibiotic resistance and related death—the estimated risk functions for lung cancer due to smoking grossly varied depending on the validity of the input data. The aggregated association between smoking and death ranged from smoking “being protective” up to smoking being a much higher risk factor than what the solid individual-level data of many cohorts already established.

Public health action must be based on scientific evidence, derived with adequate methods. In the absence of key indicators needed to estimate the burden of a public health risk factor, one should abstain from such exercises. Public health policy claims such as the call for clean air to abate deaths due to antibiotic resistance can lead to unsubstantiated changes in the One Health strategies to abate antibiotic resistance and the related burden of death and diseases. Such claims can contribute to harm instead of fostering the most efficient and urgent science based solutions, as those propagated by WHO [9].
